# Stercoral perforation of the colon: a mortal consequence of chronic constipation in the elderly (a case report)

**DOI:** 10.11604/pamj.2021.38.48.22948

**Published:** 2021-01-18

**Authors:** Bouali Mounir, Lafkih Oussama, Abbad El Andaloussi Zineb, El Bakouri Abdelilah, El Hattabi Khalid, Bensardi Fatima-Zahra, Fadil Abdelaaziz

**Affiliations:** 1 Department of Visceral Surgical Emergency, Ibn Rochd University Hospital, Casablanca, Morocco,; 2 Department of Radiology, Ibn Rochd University Hospital, Casablanca, Morocco

**Keywords:** Constipation, fecal impaction, perforation, case report

## Abstract

The stercoral perforation is a mortal condition. It affects elderly patients who have a long history of chronic and severe constipation as well as constitutes a surgical emergency whose prognosis, often grim, depends on the early diagnosis and treatment. We report the case of a stercoral colon perforation which occurred in an 89-year-old patient. The clinical symptomatology was that of an acute peritonitis evolving for four days. The diagnosis was only made intraoperatively and the surgical gesture was a resection of the involved left colon segment and Bouilly Volkmann colostomy. The consequences were unfortunately marked by a resistant septic shock resulting in the death of the patient on the 1^st^ postoperative day. The diagnosis of stercoral colon perforation, which is often difficult and delayed, must be known by all physicians who manage an increasingly older patient population.

## Introduction

Stercoral perforation is a rare condition. It mainly affects older, weak and bedridden patients. The disease was first reported in 1894 [1]. Chronic constipation is the main trigger cause. The fecal impaction on the walls of the colon causes necrotic and gangrenous ulcer, while the recto-sigmoid is the most often affected segment. Perforation is life-threatening and can be confused with other causes of acute abdomen in these patients. Early diagnosis surgery can save lives [2]. We report, the case of an 89-year-old patient man, with Alzheimer's disease and a history of chronic constipation. He had a fecal impaction which caused a sigmoid perforation with stercoral peritonitis.

## Patient and observation

**Figure 1:**
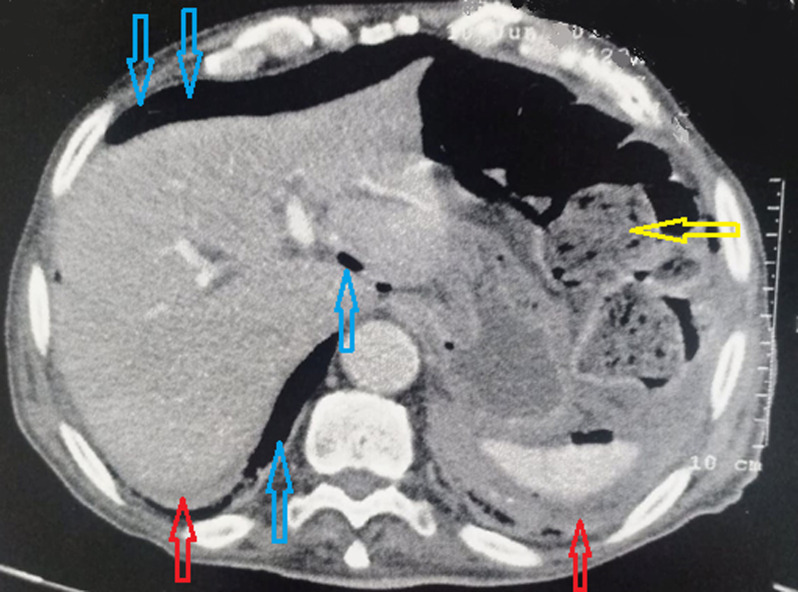
abdominal CT revealing pneumo peritoneum (blue arrow), and moderate free fluid (red arrow), and fecal impaction of the large bowel (yellow arrow)

**Figure 2:**
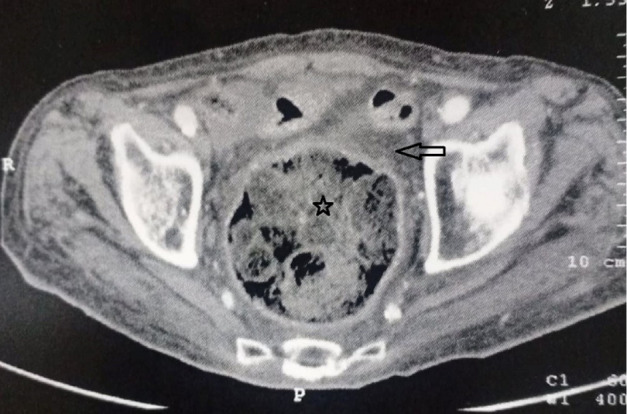
abdominal CT revealing peri-rectal fat standing (arrow) dilation of the rectum, site of several faecalomas (star)

**Figure 3:**
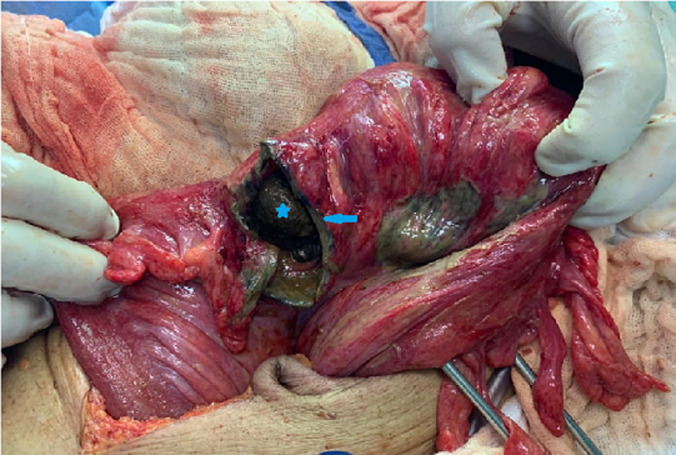
ovoid sigmoid perforation (star), on the anti-mesenteric side, measuring 5 cm in diameter, with a very hard fecal impaction (arrow) through this orifice

An 89-year-old man, with a history of chronic constipation, was admitted to the emergency room of Ibn Rochd University Hospital, as he suffered diffuse abdominal pain and rectal bleeding that had begun began 4 days earlier. He had a medical history of hypertension, Alzheimer's disease and epilepsy under valproic acid. The patient was operated for a peritonitis by perforated gastric ulcer in 2012. At the time of admission, his temperature was 38.5°C and he showed signs of hemodynamic collapse. The abdominal examination showed generalized abdominal defense more accentuated in the left iliac fossa and hypogastric area. The digital rectal examination showed a fecal impaction. While his laboratory investigations showed a biological inflammatory syndrome. The white blood cell count was 24.000 cells/mm^3^ and C-reactive protein level was 300 mg/L. The erect chest X-ray did not show a pneumoperitoneum or a hydro-aeric level. The abdominal computerized tomography (CT) (Figure 1) revealed pneumoperitoneum, with focal wall thickening of the proximal sigmoid colon and moderate free fluid in the pelvis with a megacolon, and fecal impaction of the large bowel with a peri-colic and peri-rectal fat standing (Figure 2). A diagnostic laparotomy was applied through a midline incision under general anesthesia after carrying out resuscitation measures. Surgical exploration (Figure 3) showed generalized peritonitis with presence of false membranes and murky, purulent fluid throughout the abdomen. An ovoid sigmoid perforation was found on the anti-mesenteric side, measuring 5 cm in diameter, with a very hard fecal impaction through this orifice. The sigmoid colon was resected with Bouilly Volkmann colostomy and intraperitoneal irrigation with massive amounts of warm normal saline solution and adequate placement of drainage tubes were performed. Postoperatively, he was on ventilator support and inotropes and as soon as the first day under ventilator, he developed hemodynamic instability and he died. The pathology revealed a single perforation and inflammatory changes in the sigmoid wall without signs of malignancy or specificity.

## Discussion

Stercoral perforation is a complication of chronic constipation. It is a rare but dangerous disease with an estimated mortality rate ranging from 32 to 57% [3]. It represents 3.2% of all colonic perforations. The pathogenesis is not clear, but the impacted fecaloma seems to diminish intestinal perfusion. The resulting ischemia can lead to pressure necrosis, ulceration and then perforation. The three most frequent locations of the stercoral perforation are the rectum near the peritoneal reflection, the recto-sigmoid junction and the apex of the sigmoid colon in the anti-mesenteric border [4]. The latter corresponds to the location of the stercoral perforation in our patient. Constipation is the most common symptom. It is more common in the elderly. Risk factors for constipation include extrinsic factors (narcotics, codeine, non-steroidal anti-inflammatory drugs, tricyclic antidepressants and tranquilizers), and intrinsic factors (such as diabetes, cognitive impairment, hypothyroidism, Parkinson's disease or stroke) most cases have been reported in elderly, psychiatric, bed-ridden patients with a history of constipation [5,6]. Our patient was elderly with a history of chronic constipation and had Alzheimer's disease. The symptomatology is not specific. The clinical signs are those of peritonitis by hollow abdominal organ perforation. The main symptoms are pain, fever with an acute abdominal tenderness defense or even contracture with signs of sepsis. Biological examinations show an inflammatory syndrome [5,7] which corresponds to the clinical presentation of our patient.

On imaging, free air under the diaphragm appears, on chest X-ray, in only 30% of colonic perforations [4]. The abdominal CT generally shows a focal thickening of the colonic or rectal wall. It reveals a dilation of the sigmoid colon and rectum, site of several fecalomas with peri-colic and/or peri-rectal fat stranding. The perforation is revealed by the presence of intramural or extraluminal bubble of gas or of an abscess [8]. In our case the presence of a pneumoperitoneum on the CT was the sign of perforation. According to Maurer *et al*. [9] the diagnostic criteria of stercoral perforation include: 1) a round or ovoid colonic perforation, which measures more than 1 cm in diameter located on the anti-mesenteric side; 2) the presence of fecalomas within the colon, protruding through the perforation or lying within the abdominal cavity; 3) the presence of microscopic necrosis or pressure ulcer with a chronic inflammatory reaction around the site of perforation; 4) abdominal trauma or other colonic pathology causing colonic perforations are excluded. These criteria are not useful for preoperative diagnosis. They are based on the results of surgical exploration [5]. Stercoral perforation is a rare condition that is life-threatening. It presents a surgical emergency. The treatment is based on the resection of the involved segment of the left colon and proximal colostomy. In some patients with localized intra-abdominal sepsis, segmental resection of the perforated colon with a primary anastomosis and a proximal colostomy could be done, but this is correlated to a high mortality rate [10,11]. The main factor in choosing the intervention type is patient´s safety, surgeons should not hesitate to perform an enterostomy or a Hartmann´s operation [5]. Our surgical exploration found generalized stercoral peritonitis with the four stercoral perforation diagnostic criteria. This justified segmental colonic resection with a Bouilly Volkmann colostomy.

## Conclusion

In the elderly, chronic constipation should always be treated with dietary and pharmacological measures. When mismanaged, the biggest risk is to develop ulcers and stercoral perforations. A stercoral perforation diagnosis must be highly suspected in patients with a history of chronic constipation who present with acute abdominal pain. Early surgical intervention improves the prognosis, most often bleak.

## References

[1] Berry J (1894). Dilation and rupture of the sigmoid flexure. Br Med J.

[2] Celayir MF, Köksal HM, Uludag M (2017). Stercoral perforation of the rectosigmoid colon due to chronic constipation: a case report. Int J Surg Case Rep.

[3] Poitras R, Warren D, Oyogoa S (2018). Opioid drugs and stercoral perforation of the colon: case report and review of literature. Int J Surg Case Rep.

[4] Kanwal D, Attia KME, Fam MNA, Khalil SMF, Alblooshi AM (2017). Stercoral perforation of the rectum with faecal peritonitis and pneumatosis coli: a case report. J Radiol Case Rep.

[5] Ryu CG, Kim P, Cho MJ, Shin M, Jung EJ (2017). Clinical analysis of stercoral perforation without mortality. Dig Surg.

[6] Kwag SJ, Choi SK, Park JH, Jung EJ, Jung CY, Jung SH (2013). A Stercoral perforation of the rectum. Ann Coloproctology.

[7] Mahmoudi A, Maâtouk M, Noomen F, Nasr M, Zouari K, Hamdi A (2015). La perforation stercorale du côlon: à propos d´un cas et revue de la littérature. Pan Afr Med J.

[8] Coulier B, Tilquin O, Ramboux A (2015). Massive fecal peritonitis caused by stercoral sigmoid colonic perforation in the elderly. J Belg Soc Radiol.

[9] Maurer CA, Renzulli P, Mazzucchelli L, Egger B, Seiler CA, Büchler MW (2000). Use of accurate diagnostic criteria may increase incidence of stercoral perforation of the colon. Dis Colon Rectum.

[10] Chakravartty S, Chang A, Nunoo-Mensah J (2013). A systematic review of stercoral perforation. Colorectal Dis.

[11] Marget M, Ammar H (2017). Not your usual constipation: stercoral perforation. BMJ Case Rep.

